# Integrated regulatory network reveals the early salt tolerance mechanism of *Populus euphratica*

**DOI:** 10.1038/s41598-017-05240-0

**Published:** 2017-07-28

**Authors:** Jiafei Chen, Jin Zhang, Jianjun Hu, Wenwei Xiong, Chunguang Du, Mengzhu Lu

**Affiliations:** 1 State Key Laboratory of Tree Genetics and Breeding, Key Laboratory of Tree Breeding and Cultivation of the State Forestry Administration, Research Institute of Forestry, Chinese Academy of Forestry, Beijing, 100091 China; 2 Department of Biology, Montclair State University, Montclair, NJ 07043 USA

## Abstract

Salinization is one of the major factors that threaten the existence of plants worldwide. *Populus euphratica* has been deemed to be a promising candidate for stress response research because of its high capacity to tolerate extreme salt stress. We carried out a genome-wide transcriptome analysis to identify the differentially expressed genes (DEGs) response to salt shock and elucidate the early salt tolerance mechanisms in *P*. *euphratica*. Both hierarchical clustering and DEG analysis demonstrated a predominant variation from time-course rather than NaCl intensity within 24 hours salt shock. Among the identified 1,678 salt-responsive DEGs, 74.1% (1,244) have not been reported before. We further created an integrated regulatory gene network of the salt response in *P*. *euphratica* by combining DEGs, transcription factors (TFs), *Helitrons*, miRNAs and their targets. The prominent pathways in this network are plant hormone transduction, starch and sucrose metabolism, RNA transport, protein processing in endoplasmic reticulum, etc. In addition, the network indicates calcium-related genes play key roles in *P*. *euphratica* response to salt shock. These results illustrated an overview of the systematic molecular response in *P*. *euphratica* under different intensities of salt shock and revealed the complex regulatory mechanism.

## Introduction

Salinization is thought to be one of the major factors contributing to the degradation of land and water resources that causes environmental problems and limits worldwide food crop productivity^1^. Salinity acting primarily as an osmotic stress and cause the disruption of homeostasis and ion distribution in the cell. Moreover, it causes oxidative stress, damages to membranes and proteins, and activates signaling cascades leading to changes in gene expression^2^. As a complex trait, salt tolerance is controlled by multiple genes and involves various physiological and biochemical mechanisms. Therefore, identification of salt stress response genes and elucidation of salt tolerance mechanisms are two key issues that have recently attracted considerable research attention.

During the evolution, salt-tolerant plants have evolved a series mechanisms for adaptation to stressful salinity environments. As the only arborescent species naturally distributed at the limit of barren desert or semi-barren desert worldwide, *Populus euphratica* is well known for its distinguished salt tolerance^3^. It can grow in 2% salinity soils and even survive in 5% salinity soils^4^. Recently, the whole-genome sequence of *P*. *euphratica* ^5^ provides an indispensable resource to reveal the salt-resistant mechanisms.

A series of salt-responsive genes have been identified in plants^6^, and several signaling pathways involved in response to salt stress have been predicted and confirmed in model herbaceous plants^7^. At the cellular level, the salt overly sensitive (SOS) pathway which control Na^+^ homeostasis has been established in the model plant *Arabidopsis* ^8^. The Na^+^/H^+^ antiporter *NHX7* (*SOS1*) is the first identified core gene in this pathway, SOS1 is involved directly in the transport of Na^+^ across the plasma membrane^9^. In addition, the salt-sensitive signaling networks also control cellular K^+^ and Ca^2+^ during the responses of poplar to salinity. In plant cell, cytosolic Ca^2+^ acts as a second messenger and maintains in a low level. Under salt stress, the elevated Na^+^ elicit the increase of cytosolic Ca^2+^, and further induce signaling processes that manage the plants’ response to stress. The *SOS3* gene encodes an EF-hand type calcineurin B-like calcium-binding protein. In yeast, calcineurin plays a central role in regulation of Na^+^ and K^+^ transport. SOS3 activates SOS2, a serine/threonine type protein kinase, and the SOS2-SOS3 complex enhances SOS1-mediated Na^+^ transport^10^. Transcription factors (TFs) are integral in linking salt sensory pathways to various stress tolerance responses. The members in TF families, such as bZIP, MYB, NAC, WRKY, AP2/ERF, bHLH, etc. These TFs in turn regulate the expression of various genes that may ultimately influence the level of salt tolerance of plants^11^. In addition, the transcriptional regulation of these stress-responsive genes is mediated by dynamic changes in hormone biosynthesis. After stress induction, the level of various plant hormones including abscisic acid (ABA), jasmonate (JA), gibberellic acid (GA), and brassinosteroid (BR) were affected^12^.

Previous studies showed that ionic and osmotic homeostasis played major roles for salt tolerance of *P*. *euphratica* ^2, 3^. However, the mechanism of salt tolerance is a highly sophisticated process regulated by many genes and metabolic pathways^13^, further exploration of novel regulatory pathways or diverse roles of model-plants-derived salt-responsive factors in salt-tolerant plants is quite helpful to understand the complicated regulatory mechanism underlying the salt response process of plants. An intriguing question is how woody plants, with typical features of bulk mass and long life span, respond to excess salt and develop varying strategies to adapt to high salinity. *P*. *euphratica* has been deemed to be a promising candidate for salt stress response research because of its high capacity to tolerate extremely high salinity^14, 15^.

In this study, we created the regulatory network of salt stress responses by incorporating DEGs, *cis*-regulatory elements, TFs, *Helitrons*, microRNAs and their targets and discovered that calcium is a major player in the salt related network. Our results illustrated an overview of the systematic molecular response of *P*. *euphratica* under different salt treatments and should shed some light on the complex regulatory mechanism of *P*. *euphratica* response to salt stress.

## Materials and Methods

### Plant material and salt treatment

Well-grown 1-meter-high *P*. *euphratica* seedlings individually planted in 10 L pots containing local loam soil were employed for salt treatment at Yuli, Xinjiang Uygur Autonomous Region of China (E86°17′21″, N41°19′08″). Plants in pots were completely irrigated once a week with 2 L local groundwater. For salt treatment, 2 L sodium chloride (NaCl) solution at 400 mM and 600 mM concentration, respectively, was irrigated to each potted plant and equal amount of solution without NaCl was set as control (CK). Apical buds with the top 5 young leaves were harvested at 0, 6, 12, and 24 h after salt exposure (HAE) and frozen in liquid nitrogen immediately. Tissues from three plants were mixed as one sample and three replicates were collected for each sample (i.e. a total of 9 plants per treatment were employed at each time point). To eliminate potential influences by non-salt-stress factors, samples were consistently collected at 16:00 (UTC + 8) based on different start times of salt treatment.

### RNA preparation

Frozen tissue was grinded into powder in mortar. Total RNA was extracted from 500 mg frozen plant powder using TRIzol reagent, and was further purified and concentrated with the RNeasy MinElute Cleanup kit (Qiagen Valencia, CA, USA), followed by an on-column DNase treatment. The RNA quality was verified using an Agilent Bioanalyzer (Agilent Technologies). The high-quality total RNAs were used for further microarray hybridization and following quantitative real-time PCR (qRT-PCR) validation.

### Microarray hybridization and data analysis

Approximate 2 μg of total RNA was amplified using two-cycle Affymetrix labeling method according the standard Affymetrix protocol. The Affymetrix GeneChip^®^ Poplar Genome Array was employed in this analysis, which contains 56,055 transcripts, including all UniGene clusters, ESTs and mRNAs, predicted gene transcripts, poplar control, and rRNAs from all *Populus* species with detection sensitivity 1: 100,000. The microarray data in our study has been deposited in NCBI Gene Expression Omnibus (GEO) with accession number GSE52305.Transcriptome analysis was conducted using Partek Genome Suite software (http://www.partek.com/). Data preprocessing was conducted based on the Robust Multi-array Averaging (RMA) algorithm^16^. Pearson correlation coefficients were computed on the RMA expression values (log2 transformed) for each set of biological replicates. Two-way ANOVA was performed in the form of “Y_ijk_ = μ + T_i_ + TP_j_ + (T × TP)_ij_ + ε_ijk_” where effects from treatments (T_i_), time points (TP_j_), and their interactions ((T × TP)_ij_) were considered. False discovery rate (FDR) was employed to correct from multiple comparisons^17^, and DEGs were filtered based on a joint criterion of “FDR < 0.05” and “fold change value more than 1 or less than −1 (log_2_ transformed)” between any pair-wise comparisons.

### Bioinformatic analyses

The 234 miRNAs of *P*. *trichocarpa* and 5 miRNAs of *P*. *euphratica* from miRBase (version 18)^18^, as well as published putative miRNAs of *P*. *euphratica* ^19, 20^ were collected to construct a non-redundant poplar miRNA dataset, of which putative targets were identified among the DEGs by online tool psRNATarget^21^ under the default parameters. We took the consensus sequences of the DEGs and searched against the TRANSFAC^22^ database to identify the putative TFs. The sequence of 1 kb upstream of the DEGs were downloaded from Biomart^23^ and subjected to online tool PlantCARE^24^ for the prediction of *cis*-regulatory elements. We also predicted the *Helitrons* from *P*. *trichocarpa* genome via HelitronScanner^25^, and further identified DEG-carried *Helitrons* by BLAST between predicted *Helitrons* and consensus sequences of identified DEGs. Altogether we integrated the DEGs, TFs, microRNAs, and *Helitrons* and constructed the regulatory network using Boolean logic method^26^. The graphic presentation of the integrated network based on KEGG^27^ was created by Cytoscape^28^. K-means clustering of DEGs were analyzed using MeV software^29^.

### qRT-PCR

The qRT-PCR was performed with a 7500 Real Time PCR System (Applied Biosystems) and a SYBR Premix Ex Taq^TM^ Kit (TaKaRa). Primer pairs were designed against the sequences of selected genes via Primer Express (version 3.0, Applied Biosystems) to amplify target fragments between 150 and 200 bp, and the primers employed for qRT-PCR were listed in Supplementary Table S9. The single-stranded cDNAs were synthesized from 2 μg of the same total RNA applied to microarray hybridization using the SMART cDNA Synthesis Kit (Clontech) and diluted 50-fold with RNase-free water. PCR reactions were conducted in 25-μl volumes containing 1 μl of diluted single-stranded cDNA, 1 × SYBR^®^ Premix Ex Taq^TM^ SYBR Green I Master Mix, and 0.5 μM of each primer. Six replicates were carried out in parallel for each gene, and *cyclophilin* (GenBank: AJ776859) was used as internal control.

## Results

### Transcriptional characteristics of *P*. *euphratica* response to early salt stress

**Figure 1:**
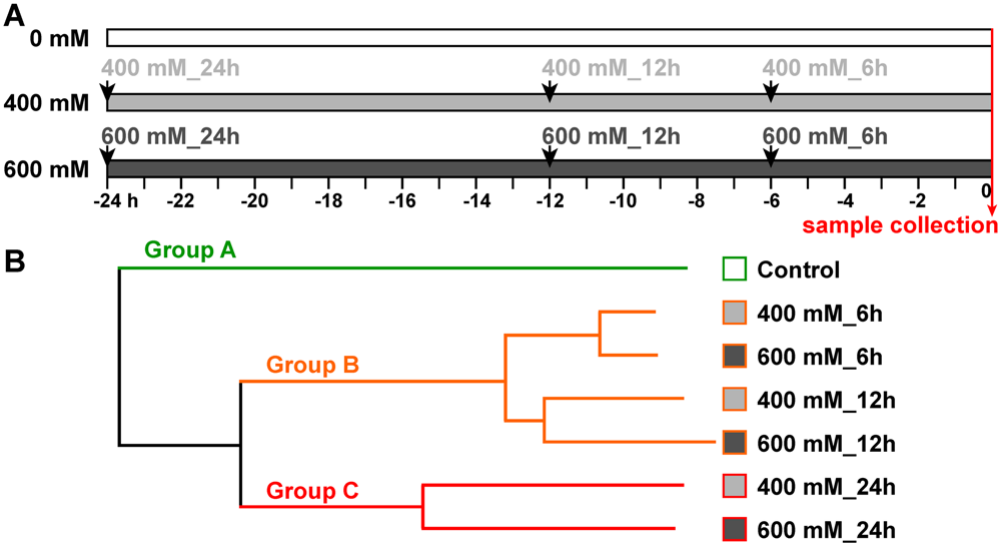
Samples collection and hierarchical cluster dendrogram of all the samples. (**A**) The seedlings were treated at 24 h, 12 h, and 6 h using 400 mM or 600 mM NaCl before samples collection. All the samples including control (0 h, without treatment) were collected at the same time to exclude the effects of circadian. (**B**) hierarchical cluster dendrogram of normalized transcript abundances from seven samples based on the complete distance linkage.

To reveal the early salt-tolerance mechanism of *P*. *euphratica*, we performed a time-course salt treatment. As shown in Fig. 1A, the *P*. *euphratica* seedlings were treated using 400 mM or 600 mM NaCl at 6 h, 12 h, and 24 h before sample collection, all the treated samples and control were collected at the same time to eliminate the potential influences by circadian or other environmental factors. Then the transcriptome of the samples from the six time points during the salt stress (400 mM_6 h, 400 mM_12 h, 400 mM_24 h, 600 mM_6 h, 600 mM_12 h, and 600 mM_24 h) and control (Ctrl) were analyzed using microarray. For microarray assay, each time point had two replicates.

After normalization, the expression patterns of all the transcripts from the seven time points were used for hierarchical cluster analysis. Based on the global gene expression pattern, the seven samples were classed into three main groups (A, B, and C). Group B was composed by four samples (400 mM_6 h, 600 mM_6 h, 400 mM_12 h, and 600 mM_12 h) and group C was composed by two samples (400 mM_24 h and 600 mM_24 h). The control sample (group A) was significantly divergent from all the samples treated under salt stress at transcriptional level. Interestingly, the samples treated using both 400 mM and 600 mM NaCl were clustered together in each time point, indicating the transcriptional responses of *P*. *euphratica* to 400 mM and 600 mM NaCl showed similar trends during early stress stages (in 24 h). In addition, the samples treated after 6 h and 12 h under both 400 mM and 600 mM NaCl were clustered together closely, and were significant different from the samples treated after 24 h; indicating that the transcriptional response of *P*. *euphratica* trend to a relative stable state during 6 h to 12 h no matter under 400 mM or 600 mM NaCl treatment (Fig. 1B). Hierarchical clustering of the transcriptional data demonstrates a predominant variation trend from time course rather than salt intensity in *P*. *euphratica* early response to salt stress.

To screen authentically stress-responsive genes, the differentially expressed genes (DEGs) were identified based on the criteria “log_2_ transformed fold change ≥1 or ≤−1” and “FDR < 0.05”. As shown in Table 1, more than 4,000 DEGs were identified during the early salt stress stages under 400 mM or 600 mM NaCl when compared with control. We also compared the time-course DEGs between the neighbor time points. Interestingly, the transcriptome showed high similarity between 6 h and 12 h, there are only 32 DEGs and 85 DEGs were identified in the comparisons “400 mM_12 h vs. 400 mM_6 h” and “600 mM_12 h vs. 600 mM_6 h”, respectively. In addition, more DEGs were identified under 600 mM NaCl stress rather than 400 mM NaCl between 12 h and 24 h.

**Table 1:** DEGs identification in *P*. *euphratica* response to salt stress.

	Up-regulated genes	Down-regulated genes	Total DEGs
400 mM_6 h vs. Ctrl	2184	2407	4591
400 mM_12 h vs. Ctrl	2659	2884	5543
400 mM_24 h vs. Ctrl	2446	2693	5139
400 mM_12 h vs. 400 mM_6 h	17	15	32
400 mM_24 h vs. 400 mM_12 h	687	528	1215
600 mM_6 h vs. Ctrl	2675	2888	5563
600 mM_12 h vs. Ctrl	2579	2541	5120
600 mM_24 h vs. Ctrl	2313	2460	4773
600 mM_12 h vs. 600 mM_6 h	40	45	85
600 mM_24 h vs. 600 mM_12 h	1411	1427	2838

**Figure 2:**
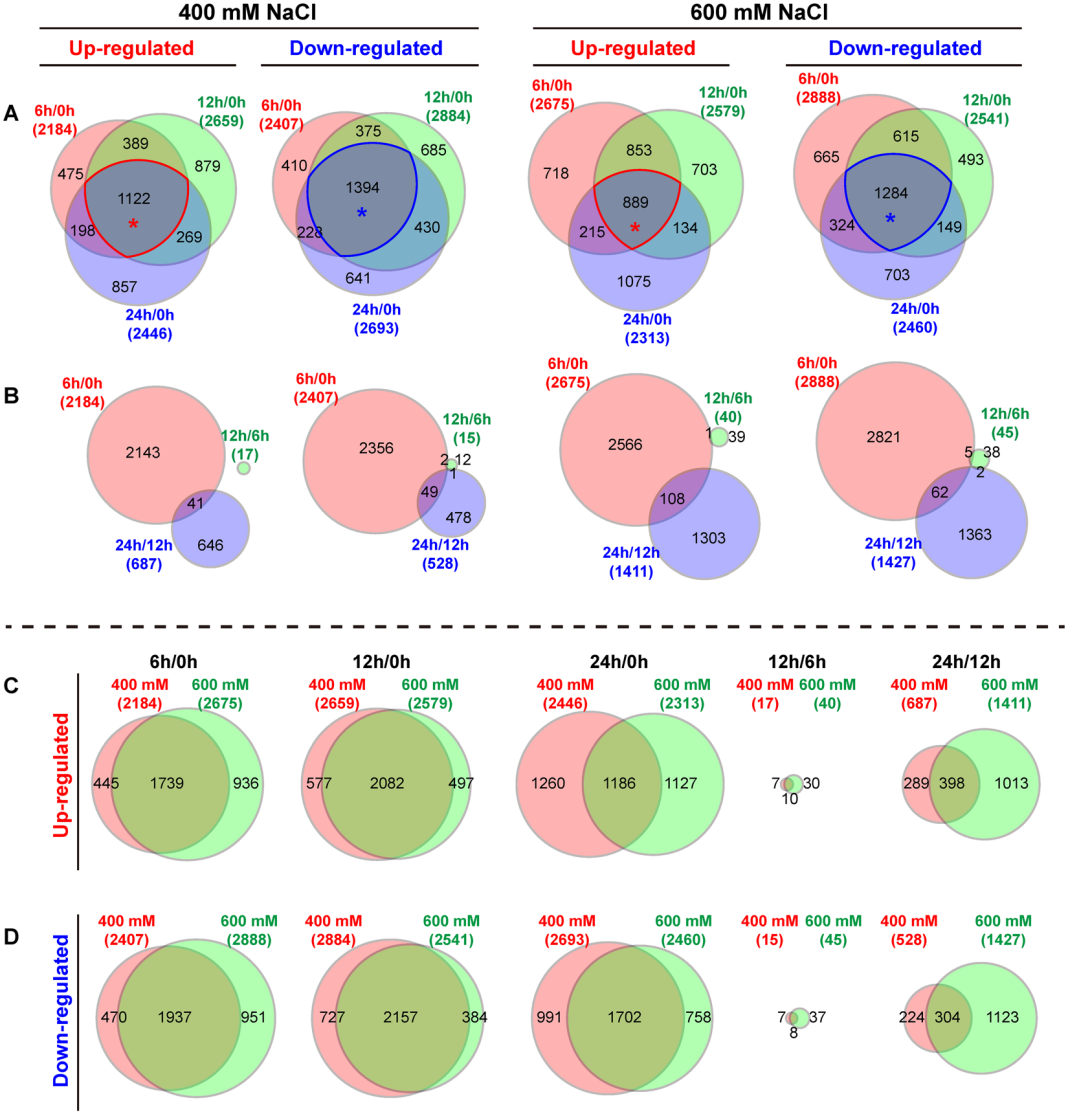
Venn diagrams show different comparisons of *P*. *euphratica* during salt stress. (**A**) Overlap of comparison 6 h/0 h, 12 h/0 h, and 24 h/0 h of up- or down-regulated genes after 400 mM or 600 mM NaCl treatment. Red and green asterisks indicate the overlapped up- and down-regulated core genes in the three comparisons. (**B**) Overlap of comparison 6 h/0 h, 12 h/6 h, and 24 h/12 h of up- or down-regulated genes after 400 mM or 600 mM NaCl treatment. (**C**) Overlap of up-regulated genes between 400 mM and 60 mM NaCl treatments in five comparisons (6 h/0 h, 12 h/0 h, 24 h/0 h, 12 h/6 h, and 24 h/12 h). (**D**) Overlap of down-regulated genes between 400 mM and 60 mM NaCl treatments in five comparisons (6 h/0 h, 12 h/0 h, 24 h/0 h, 12 h/6 h, and 24 h/12 h). The circular area corresponding to the gene number in each comparison.

To confirm the core genes in *P*. *euphratica* response to early salt stress, we analyzed the overlapped genes among different comparisons. Under 400 mM NaCl stress, total of 1,122 up-regulated genes and 1,394 down-regulated genes were overlapped among the comparisons “400 mM_6 h vs. Ctrl”, “400 mM_12 h vs. Ctrl”, and “400 mM_24 h vs. Ctrl”. While the overlapped genes were smaller under 600 mM NaCl stress than 400 mM NaCl stress, total of 889 up-regulated genes and 1,284 down-regulated genes were overlapped among the comparisons “600 mM_6 h vs. Ctrl”, “600 mM_12 h vs. Ctrl”, and “600 mM_24 h vs. Ctrl” (Fig. 2A). This indicated that the divergence among the three time points were larger under 600 mM than 400 mM, although the genome-wide relationships were smaller in Fig. 1. By contrast, no up- or down-regulated genes were overlapped in the time-course comparisons “6 h vs. Ctrl”, “12 h vs. 6 h”, and “24 h vs. 12 h” under both 400 mM and 600 mM NaCl treatments. On one hand, few DEGs were identified between 6 h and 12 h. On the other hand, fewer DEGs were overlapped between “6 h vs. Ctrl” and “24 h vs. 12 h”; only 41 up-regulated and 49 down-regulated genes were overlapped in the two comparisons under 400 mM NaCl stress and 108 up-regulated genes and 62 down-regulated genes were overlapped under 600 mM NaCl stress (Fig. 2B). This implied different DEGs involved divergent molecular response to corresponding stage during the time-course salt stress.

In addition, we analyzed the overlapped genes between the comparisons under 400 mM and 600 mM NaCl treatments. As shown in Fig. 2C and D, most of the up- or down-regulated genes in comparisons “6 h vs. Ctrl” and “12 h vs. Ctrl” were overlapped under 400 mM and 600 mM NaCl treatments. There were 1,739 up-regulated genes and 1,937 down-regulated genes overlapped between “400 mM_6 h vs. Ctrl” and “600 mM_6 h vs. Ctrl”, and 2,082 up-regulated genes and 2,157 down-regulated genes were overlapped between “400 mM_12 h vs. Ctrl” and “600 mM_12 h vs. Ctrl”. In contrast, the ratio of overlapped DEGs in “400 mM_24 h vs. Ctrl” and “600 mM_24 h vs. Ctrl” were relative low, only 1,186 up-regulated genes and 1,702 down-regulated genes were overlapped in the two comparisons. In time-course comparisons, 10 up- and 8 down-regulated genes were overlapped in “400 mM_12 h vs. 400 mM_6 h” and “600 mM_12 h vs. 600 mM_6 h”; 398 up- and 304 down-regulated genes were overlapped in “400 mM_24 h vs. 400 mM_12 h” and “600 mM_24 h vs. 600 mM_12 h”.

### Core DEGs identification and GO enrichment analysis

**Figure 3:**
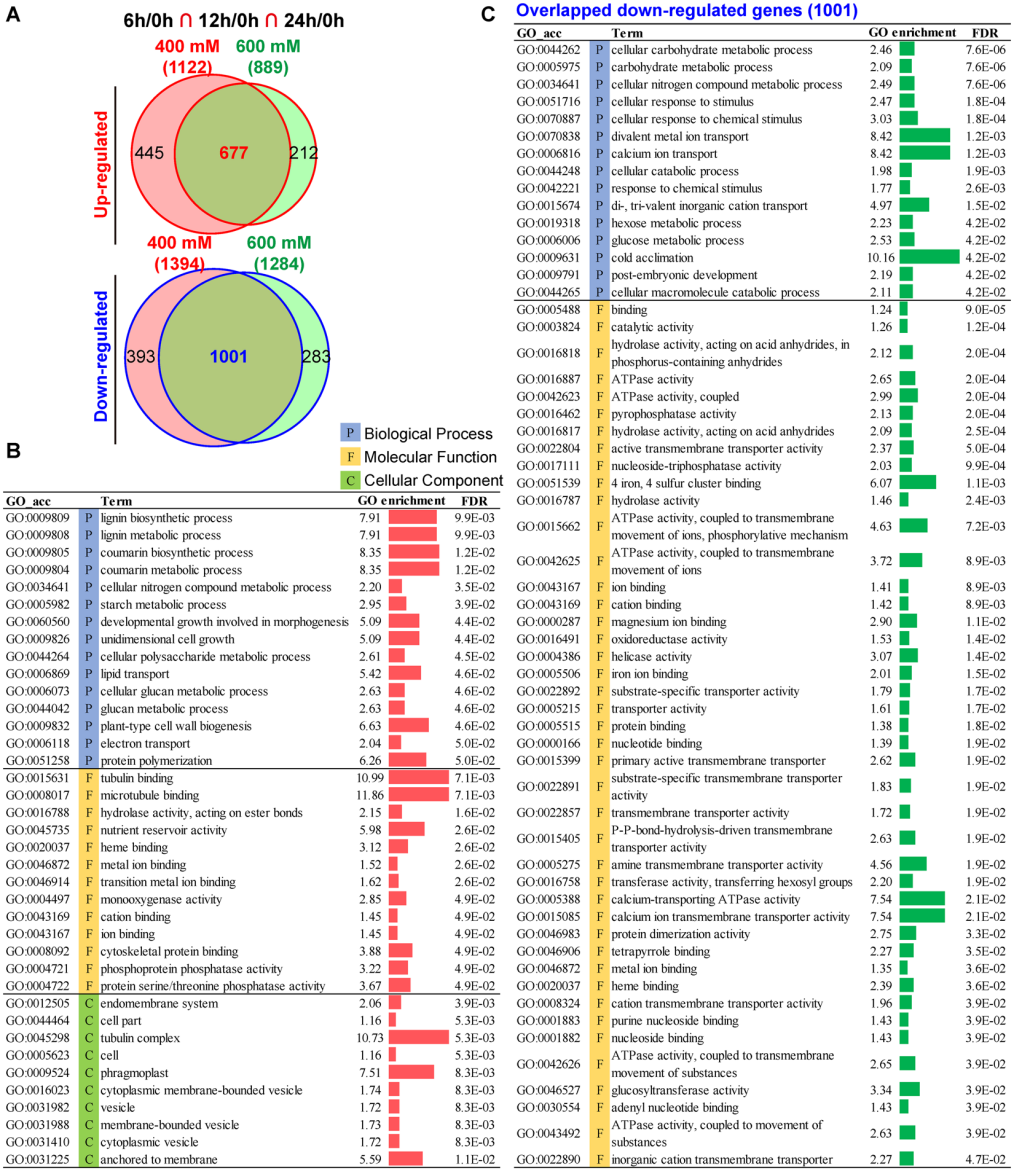
GO enrichment of up- or down-regulated core genes in *P*. *euphratica* after both 400 mM and 600 mM NaCl treatments. (**A**) Overlap of up- or down-regulated core genes in Fig. 2A between 400 mM and 600 mM NaCl treatments. (**B**) GO enrichment of 677 up-regulated core genes in both 400 mM and 600 mM NaCl treatments. (**C**) GO enrichment of 1001 down-regulated core genes in both 400 mM and 600 mM NaCl treatments.

From the overlap analysis, we identified 1,122 and 889 genes up-regulated (1,394 and 1,284 genes were down-regulated) in all the three time points (6 h, 12 h, and 24 h) compared to control under 400 mM and 600 mM NaCl stress, respectively (Fig. 2A). To further confirm the core DEGs involved in *P*. *euphratica* response to salt stress and reveal the potential functional roles of these core DEGs, we compared the overlapped genes in all these comparisons. As shown in Fig. 3A, total of 667 up-regulated genes and 1,001 down-regulated genes were overlapped. GO enrichment analysis was employed to explore the potential involved functions of the core DEGs. Based on the biological process (BP) of GO enrichment analysis, the 667 up-regulated core genes were enriched in “lignin biosynthetic process” (7.91-fold), “coumarn biosynthetic process” (8.35-fold), “developmental growth involved in morphogenesis” (5.09-fold), “lipid transport” (5.42-fold), “plant-type cell wall biogenesis” (6.63-fold), and “protein polymerization” (6.26-fold); while the 1,001 down-regulated core genes were enriched in “calcium ion transport” (8.42-fold), “cold acclimation” (10.16-fold), “cellular response to stimulus” (2.47-fold), and “di-, tri-valent inorganic cation transport” (4.97-fold). For molecular function (MF), up-regulated core genes were enriched in “microtubule binding” (11.86-fold), “nutrient reservoir activity” (5.98-fold), “cytoskeleton protein binding” (3.88-fold), and “protein serine/threonine phosphatase activity” (3.67-fold), while down-regulated core genes were enriched in “calcium ion transmembrane transporter activity” (7.54-fold), “4 iron, 4 sulfur cluster binding” (6.07-fold), “ATPase activity, coupled to transmembrane movement of ions, phosphorylative mechanism” (4.63-fold), and “glucosyltransferase activity” (3.34-fold). For cellular component (CC), up-regulated core genes were enriched in “tubulin complex” (10.73-fold), “phragmoplast” (7.51-fold), and “anchored to membrane” (5.59-fold), while no CC terms were enriched from the down-regulated core genes (Fig. 3B and C).

### K-means clustering of salt response DEGs in *P*. *euphratica*

**Figure 4:**
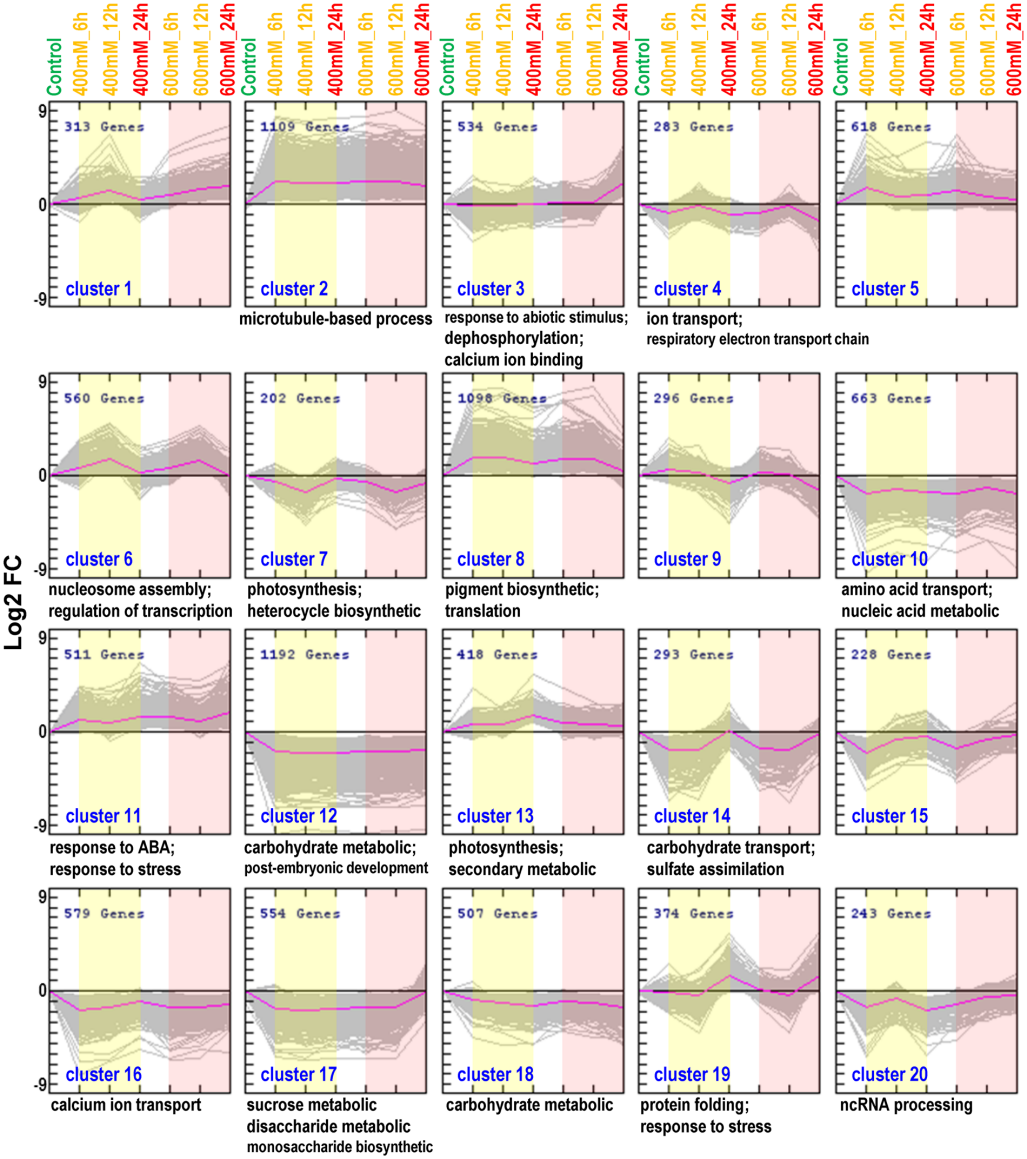
K-means clustering of DEGs in *P*. *euphratica* response to salt stress (K = 20 clusters). GO enrichments of DEGs in the 20 clusters are shown in Supplementary Table S3.

To further reveal the functional divergence of DEGs with different expression patterns in response to salt stress, the DEGs in the three time points (6 h, 12 h, and 24 h) and the two conditions (400 mM and 600 mM) were clustered via the K-means approach. From a total of 20 clusters (Fig. 4), DEGs in cluster 2 (1109 genes), cluster 8 (1098 genes), and cluster 11 (511 genes) were up-regulated under all the salt stress conditions. Moreover, DEGs in cluster 11 had relative higher expression at 24 HAE while DEGs in cluster 8 expressed relative lower at 24 HAE. Based on the GO classification, the DEGs were enriched in “microtubule-based process”, “pigment biosynthetic” and “translation”, “response to ABA” and “response to stress” in cluster 2, 8, and 11, respectively (Supplementary Table S3). DEGs in cluster 6 gradually increased at 6 and 12 HAE and then back to control level at 24 HAE under both 400 and 600 mM NaCl; these genes were enriched in “nucleosome assembly” and “regulation of transcription”. In contrast, DEGs in cluster 7 gradually decreased at 6 and 12 HAE and then back to control level at 24 HAE under 400 and 600 mM, they were enriched in “photosynthesis” and “heterocycle biosynthetic process”. In addition, DEGs in two clusters (3 and 19) were highly induced at 24 HAE. More specifically, DEGs in cluster 19 were generally up-regulated at 24 HAE under 400 and 600 mM, they were enriched in “protein folding” and “response to stress”, while DEGs in cluster 3 were specific up-regulated at 24 HAE under 600 mM NaCl, they were related to “response to abiotic stimulus”, “dephosphorylation”, and “calcium ion binding” (Fig. 4 and Supplementary Table S3).

### Regulatory network underlying the co-regulated genes in *P*. *euphratica* response to salt stress

Based on the functional classification of the Kyoto Encyclopedia of Genes and Genomes (KEGG) database^27^, a comprehensive network of pathways related with the 1678 core DEGs (677 up-regulated core genes and 1001 down-regulated core genes) was constructed. A canonical pathway mainly comprises of proteins (especially enzymes), small compounds of metabolites, and the regulations among them. A well-known fact is that TFs play a major role in regulating plants adapting to external stress, so the TFs were also incorporated into the network from the computational prediction of transcription factor binding sites (TFBS) in gene *cis*-regulatory regions by the TRANSFAC database^22^.

**Figure 5:**
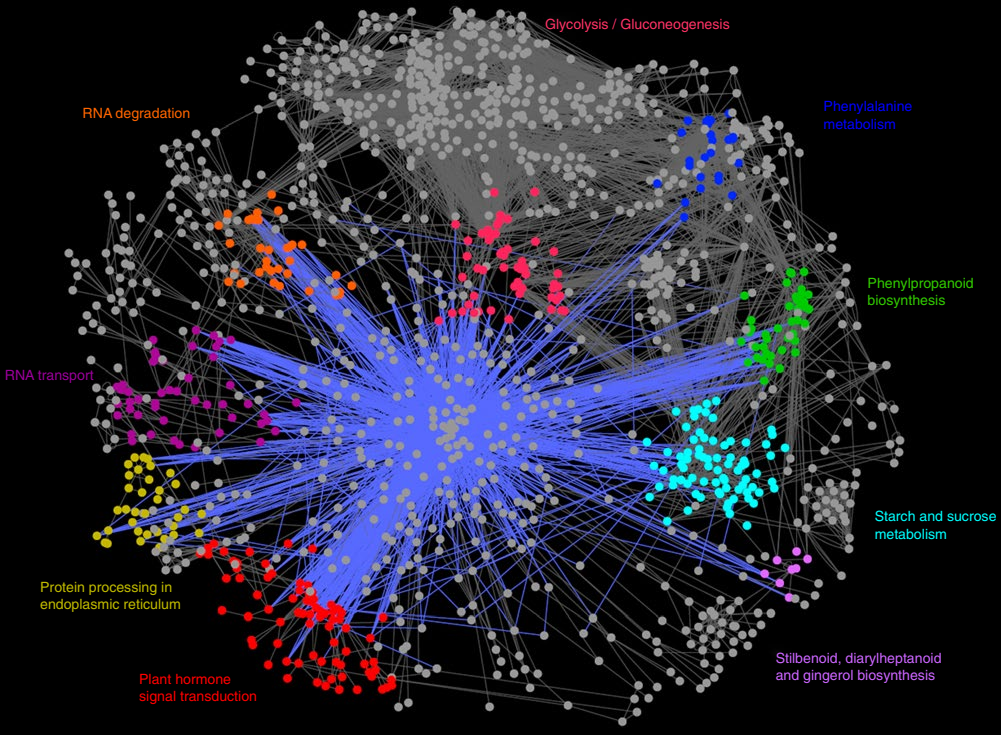
Regulatory network underlying the co-regulated genes in *P*. *euphratica* response to salt stress. Nodes involved in nine prominent pathways are marked in different colors. The blue edges indicate TFs binding relationships.

Resultantly, nine prominent pathways, including “plant hormone transduction”, “phenylpropanoid biosynthesis”, “starch and sucrose metabolism”, “RNA transport”, “protein processing in endoplasmic reticulum”, “glycolysis/gluconeogenesis”, “RNA degradation”, “phenylalanine metabolism”, as well as “stilbenoid, diarylheptanoid and gingerol biosynthesis,” which involve relatively large number of genes and TFs, are marked in colors, with the pathway names aside in same colors respectively (Fig. 5). The nodes and lines in gray are from other pathways with few genes involved. Among the above nine functional categories, the most well-represented one is the plant hormone signal transduction.

**Figure 6:**
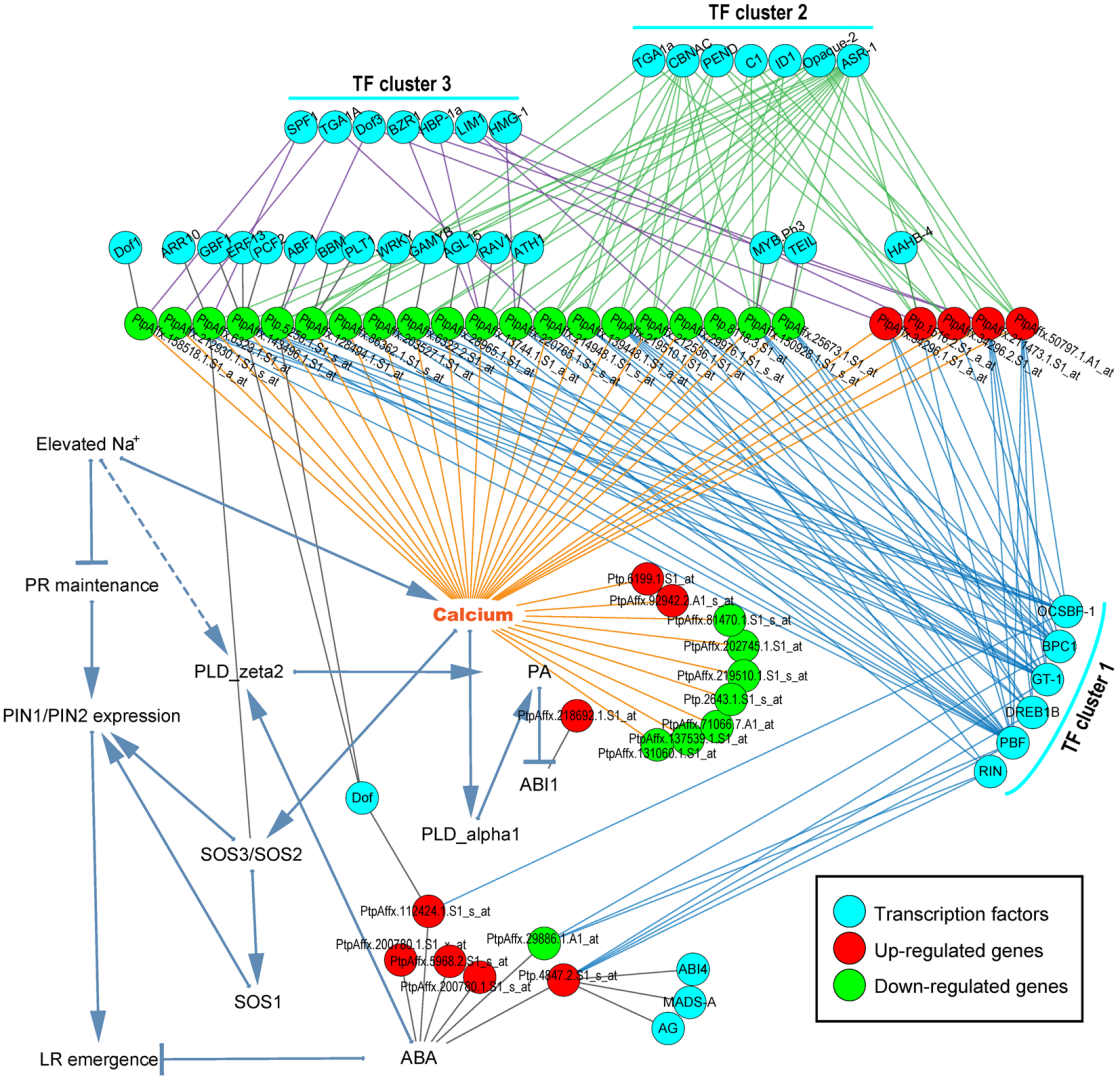
Integrated salt response pathway network in *P*. *euphratica*. Dark blue arrows (active or repress) indicate known salt-response pathways in plants, other edges indicate co-expression relationships of the up- or down-regulated core genes connected with the Calcium (Ca^2+^) or ABA signal in the known pathways. Cyan nodes, TFs; red nodes, up-regulated core genes; green nodes, down-regulated core genes. Based on the relationships between identified TFs and up- or down-regulated core genes, three main TF clusters (1, 2, and 3) were identified. TF cluster 1 strongly connected with Ca^2+^ related up- or down-regulated core genes and might be involved in ABA-dependent pathway, TF cluster 2 strongly connected with Ca^2+^ related up- or down-regulated core genes but didn’t connected with ABA pathway, while TF cluster 3 weakly connected with several Ca^2+^ related up- or down-regulated core genes.

Based on the GO enrichment analysis in Fig. 3, the biological process (BP) related with calcium ion (Ca^2+^) transport were significantly enriched in down-regulated genes (8.42-fold). As an important secondary messenger, Ca^2+^ plays crucial role in plant response to various stresses. We then constructed an integrate signal transduction-transcriptional regulatory network based on the known Ca^2+^ signal transduction and the regulatory network from Fig. 5. As shown in Fig. 6, the dark blue arrows (active or repress) connected the known Ca^2+^ signal transduction in response to salt stress and the other edges connected our study identified up-regulated genes (red nodes), down-regulated genes (green nodes), or the TFs (cyan nodes) with the known Ca^2+^ signal transduction network. According to previous study^30^, when plants percept the elevated Na^+^ under salt stress, Ca^2+^ and ABA signal cooperated to regulate a series molecular and physiological responses through SOS pathway. Based on the relationships between TFs and up-/down-regulated genes connected with Ca^2+^ or ABA, the TFs were classified into three main clusters: TFs in cluster 1 have strong correlation with the DEGs connected with both Ca^2+^ and ABA, TFs in cluster 2 have strong correlation with DEGs connected only with Ca^2+^, and TFs in cluster 3 have weak correlation with DEGs connected with Ca^2+^.

**Figure 7:**
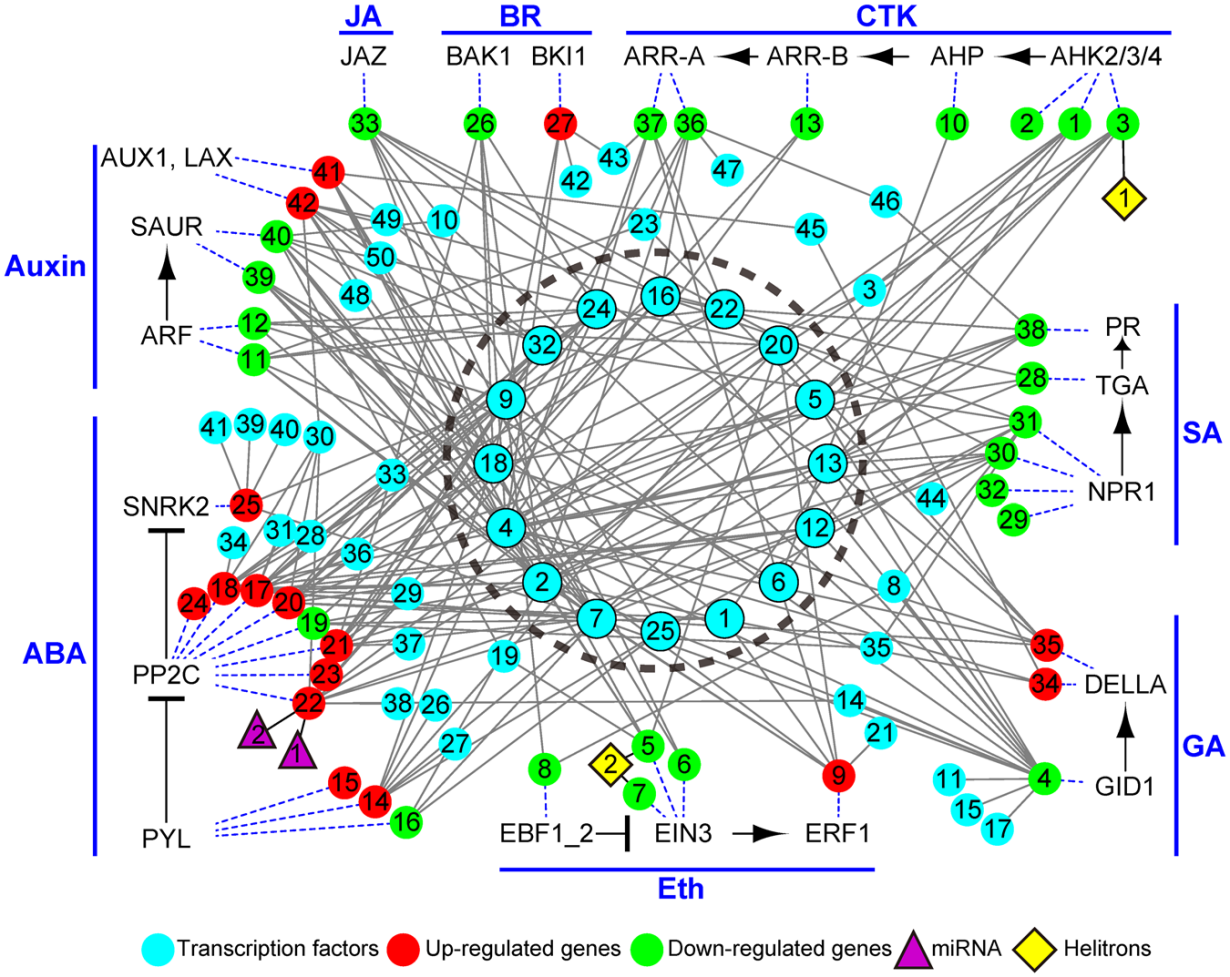
Integrated hormone signal pathway network of *P*. *euphratica* after salt stress. Cyan nodes, TFs; red nodes, up-regulated core genes; green nodes, down-regulated core genes; purple triangle, miRNA; yellow diamond, *Helitrons*. The 16 TFs in center dashed circle are widely related with multiple hormone signal pathways. Blue dash lines indicate the up- or down-regulated core genes corresponding to the genes in various hormone signal pathways. The detail of genes was shown in Supplementary Table S5.

In the network constructed based on the core DEGs, plant hormone transduction related genes were most abundant. To reveal the potential hormone regulatory mechanism of *P*. *euphratica* response to salt stress, we constructed an integrated hormone-transcriptional regulatory network. As shown in Fig. 7, the up- or down-regulated core DEGs (red or green nodes) were distributed in auxin, ABA, Eth, GA, SA, CTK, BR, and JA hormone pathways. Interestingly, most core DEGs involved in ABA pathway were up-regulated, while the DEGs involved in other hormone pathways main were down-regulated. This indicates that *P*. *euphratica* might enhance its salt tolerance through the ABA-dependent pathway. Based on these hormone-related core DEGs, we identified several TFs (cyan nodes) might be involved in their transcriptional regulation. Noticeably, a total of 16 TFs (cyan nodes in the circle dash line cycle, Supplementary Table S5) showed significant connection with multiple hormone pathways, which crosstalk to response the salt stress. In addition, miRNA and *Helitrons* were also involved in the regulatory network. PP2C genes in the ABA pathway were the targets of two microRNAs (peu-mir-155a and peu-mir-155b). Two *Helitrons* (Hel_peu_1 and Hel_peu_2) capturing the histidine kinase 3 in the CTK pathway and ethylene-insensitive 3 in the Eth pathway, respectively.

### Novel salt stress-responsive genes identified from *P*. *euphratica* in our study

In previous studies, many salt stress responsive genes were identified from several species of *Populus*. To explore the potential mechanism of distinguished salt tolerance of *P*. *euphratica*, we compared the DEGs identified in our study with previous studies. As shown in Supplementary Table S8, there are about 34.6% to 75% of the salt-stress responsive genes in *P*. *euphratica* identified from previous studies^2, 3, 31–33^. The fact that, compared to the salt sensitive poplars, salt tolerant *P*. *euphratica* possessed consistent higher expression of genes responsible for osmotic adjustment, ion compartmentalization and detoxification of reactive oxygen species^33, 34^. Nearly three-quarters of the common DEGs between Janz *et al*.^34^ and our results exhibited higher basal activity in *P*. *euphratica* than the salt-sensitive *P*. × *canescens* (Supplementary Table S8 “Janz_2010”). Interestingly, 56% to 60% of the DEGs identified to respond to varying drought intensities^35^ also appeared in our filtered list (Supplementary Table S8 “Yan_2012”), indicating an underlying cross-talk between the regulatory network of *P*. *euphratica* response to salt and drought stresses.

Even though, more differentially co-regulated transcripts, especially 401 up- and 843 down-regulated in a continuous manner in both treatments within the 24 HAE, were not yet disclosed by aforementioned publications, making it possible to facilitate the exploring of regulatory mechanism of *P*. *euphratica* response to salinity and other abiotic stresses in a wide scope. For example, transcripts homologous to AHP5 (histidine-containing phosphotransfer factor 5), ARR9 (response regulator 9), EIN3 (ethylene-insensitive3) and mass of other genes that were proved to be involved in the hormone signal transduction pathway in *Arabidopsis* exposed to abiotic stress were also found to possibly participate in stress response of this salt-tolerant poplar. Furthermore, this result was ulteriorly reinforced with the fact that partial identified DEGs possessed high-confidence matches when compared to the public *P*. *euphratica* EST database.

Previous studies have proven that most of the known *P*. *trichocarpa* miRNAs were broadly employed in congeneric species to respond to adverse environments, such as salt^36, 37^, drought^19, 20, 36, 38, 39^, and other stresses^36, 40–42^. In order to explore potential targets of stress-related miRNAs among the DEGs in this study, we collected known poplar miRNAs from database (miRBase 18.0, 234 microRNAs from *P*. *trichocarpa* and 5 from *P*. *euphratica*) and candidate miRNAs of *P*. *euphratica* from publications^19, 20^, constituting a non-redundant dataset of 479 poplar miRNAs. Among the 11,705 differentially expressed transcripts, 366 non-redundant transcripts were identified as putative targets of 331 miRNAs with a variety of correspondence (Supplementary Table S6). For instance, two transcripts encoding pentatricopeptide repeat (PPR), authorized targets of miR474 family under saline stress proved by Zhou *et al*.^37^, as well as other confirmed targets of several miRNA families (Supplementary Table S7), were found to be induced or repressed at varying levels in our investigations. Specially, 9 and 13 predicted targets showed continuous up- and down-regulated profiling in both salt intensities within 24 HAE, suggesting important roles of these microRNA-targets in the regulatory network of *P*. *euphratica* response to salt shock.

### Validation of microarray data using qRT-PCR

**Figure 8:**
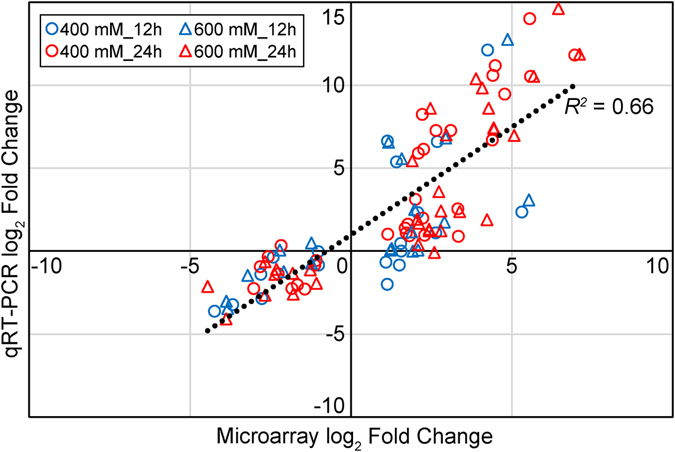
Validation of microarray data using qRT-PCR. Total of 38 genes were selected for qRT-PCR validation. The samples treated after 12 h and 24 h using 400 mM or 600 mM NaCl were compared with control. *x*-axis indicates log2 fold changes from microarray data and *y*-axis indicate log2 fold changes from qRT-PCR results. The coefficient of determination (*R* ^2^) between microarray results and qRT-PCR results was 0.66.

To validate the accuracy of our microarray data, the transcript levels of selected 38 genes at 12 and 24 HAE under 400 and 600 mM treatments were analyzed by qRT-PCR. Although the actual quantitative values differed considerably between the microarray and qRT-PCR, the same trend was observed for the 38 genes in at least two inspected time points. The results between qRT-PCR and microarray data showed a high validation rate (91.35%) and a high coefficient of determination (*R* ^2^ = 0.66) (Fig. 8). For example, the microarray data showed the transcript encoding *histone H1* (Potri.010G076800) was strongly up-regulated at 12 HAE compared to CK, and down-regulated at 24 HAE in both treatments. This was strictly validated by the qRT-PCR in both treatments (Supplementary Table S9). The similar trends of the transcriptional dynamics obtained by both microarray and qRT-PCR suggest that the transcriptional profiles of these genes within a 24-h salt response are truly reflected by the microarray data.

## Discussion

Analysis of DEGs demonstrated that *P*. *euphratica* recruited thousands of genes respond to the salt shock. The salt response DEGs identified in this study were more than that from previous studies. This could be attributed to the less designed transcripts on the employed arrays^2, 3, 31, 32^, special focus on a few gene families^33^, or just direct comparison between *P*. *euphratica* and salt-sensitive poplars without stress^33, 34^. Here we analyzed the response of *P*. *euphratica* to salt shock (short term salt stress within 24 h) under 400 mM and 600 mM NaCl. Based on the microarray data, the transcriptome showed high similarity between 6 and 12 h under both 400 mM and 600 mM NaCl treatments. The results implied the cellular response will have a relative stable state during 6–12 h in *P*. *euphratica* response to 400 mM and 600 mM NaCl.

Apart from the high reproducibility of salt-responsive genes in *P*. *euphratica* identified in this study compared to the previous studies, high degree of overlap also existed between the DEGs identified to be involved in response to varying drought stresses^35^ and salt shock. Similar results were also found in *Arabidopsis*, the genes responding to salt, cold, hormone treatment and other stress showed a large fraction of overlap^43^, these genes might play roles in multiple stress responses. In *P*. *euphratica*, this could be deciphered by the fact that this salt tolerant poplar is mainly distributed in arid and semiarid regions of central Asia, in which salt, drought, even heat stress often coexist simultaneously^44–47^, in that case it may employ the same genes but develop different regulatory mechanism to respond to the multiple stresses^48^. For example, the induction of ABA accumulation-related gene under both salt and drought stress is in line with the previous observations that it introduced increasing endogenous ABA content to respond the salt stress in *P*. *euphratica* ^49^, indicating accordance of the important cross-talk role of ABA in plants response to adverse stress^8, 50^. Qiu *et al*.^51^ analyzed the DEGs response to salt stress (100 mM NaCl) in *P*. *euphratica* callus and found numerous DEGs involved in ABA regulation and biosynthesis. This study classified the DEGs using K-means clustering method. The up-regulated DEGs in our cluster 11 were enriched in ‘response to ABA’ and ‘response to stress’. This indicates the ABA-dependent pathway plays dominant role in *P*. *euphratica* response to salt stress.

Of the nine remarkable pathways (Fig. 5), plant hormone signal transduction network is the best-represented subnetwork, among which members from PP2C family seem to be part of the key nodes. The full presences of the double negative regulatory system of ABA response (PYR/PYL/RCAR, PP2C, and SnRK2) suggested their central function in sensing signaling and transport of ABA in *P*. *euphratica* under salt stress^52^. Our study also predicted that a PP2C member, which is homolog of *Arabidopsis AHG3*, could be putatively regulated by the two microRNAs, peu-mir-155a and peu-mir-155b. Apart from ABA, the transcripts included in this subnetwork are also widely involved in the signal regulatory pathway of auxin, BR, cytokinin, ethylene, GA, JA and SA, and these DEGs were regulated by a series core TFs (Fig. 7), indicating an underlying complicated cross-talk between the regulatory networks of plant hormones in *P*. *euphratica* response to salt stress. Moreover, the fact that two transcripts homologous to *Arabidopsis AHK3* (AT1G27320) and *EIN3* (AT3G20770) were found to be captured by two identified *Helitrons* respectively, is in accordance with the possible involvement of transposable elements in the translocation of stress-response related genes in the evolutionary history of plants^53^.

Generally, TFs are thought to be the most key nodes in the regulation of gene expression. In our predicted network, *PBF*, *ASR-1*, *GT-1*, and *BPC1* were inferred to potentially regulate over 100 genes each. Dof family member *PBF* was originally found to express in the developing endosperm of grains where it is involved in the activation of seed storage protein genes, and may play role in the regulation of gibberellin-responsive genes^54^. Unclassified *ASR* is positioned within the signaling cascade of interactions among glucose, ABA, and GA^55^. Trihelix-family member *GT-1* plays a role in pathogen- and salt-induced expression of Ca^2+^-binding protein calmodulin that mediates cellular Ca^2+^ signals in response to a wide array of stimuli in higher eukaryotes^56^. Plant specific BBR/BPC family member *BPC1* were found to bind to GA, induce conformational changes in the regulatory region of the homeotic gene SEEDSTICK, and mediate MADS domain complex binding to the DNA for tissue-specific expression of target genes in *Arabidopsis* ^57^. The wide involvement of these representative TFs further implies their important roles in mediating the comprehensive response process of *P*. *euphratica* to salt shock.

Genes involved in balance of sodium and potasium were the major subject in the previous studies for their direct roles in the response process to salt stress^33, 58–60^. However, transcripts related to calcium are the most widely represented part of genes related to balance of salt ions in our predicted network. The functions of these calcium-relevant transcripts are generally involved in calcium transporting, calcium- and calmodulin binding, and CIPK-interacted. Previous studies have proved that calcium mediates root K^+^/Na^+^ homeostasis in poplar species differing in salt tolerance^61^ and members from calcium-related families, such as Calcineurin B-Like (CBL) family and CBL-interacting protein kinases (CIPK) family, have been investigated in *Populus* ^62, 63^. Recently, members from calcium-related gene families in *P*. *euphraitca*, such as CBL, have been proved to be positive in enhanced tolerance in *Arabidopsis* and *Populus* to abiotic stress^64–66^. The fact that most of the calcium-related transcript were repressed is in line with the previous study that Na^+^ adaptation required suppression of calcium-related signaling pathways^31^. For instance, transcript homologous to *Arabidopsis SOS3*, a calcium sensor that is essential for K^+^ nutrition, K^+^/Na^+^ selectivity, and salt tolerance, predicted putative targets of ARR10, ASR-1, GT-1, and SPF1 in our predicted regulatory network, was found to be down-regulated in our test regimes. As expected, further experiments are needed to confirm the concrete regulatory mechanisms of these TFs in *P*. *euphratica* response to salt shock.

## Conclusion

In this study, we provided an overview of the systematic molecular response in *P*. *euphratica* under different intensities of salt shock by microarray strategy. Within this employed system, both hierarchical cluster and screening of differentially expressed genes demonstrated a predominant variation from time course rather than salt intensity in *P*. *euphratica* response to the varying degrees of heavy salt shock. The extensive comparison with published transcriptional profiling of *P*. *euphratica* under salt or other stresses not only confirmed the effectiveness of our result, but also provide more candidates with possible roles in multiple stresses. Combined with salt-responsive elements identified in here and the results from previous studies, underlying regulatory network involved of hormones, TFs, *Helitrons*, miRNAs and their targets was constructed and elaborated. Our regulatory network indicates calcium-related genes play key role in the salt response of *P*. *euphratica*.

## Electronic supplementary material

Supplementary

Table S1

Table S2

Table S3

Table S4

Table S5

Table S6

Table S7

Table S8

Table S9
