# Supplementary material for: Integrated regulatory network reveals the early salt tolerance mechanism of Populus euphratica
Source: Sci Rep. 2017 Jul 28;7:6769. doi: 10.1038/s41598-017-05240-0 (PMC5533726; doi:10.1038/s41598-017-05240-0)
Supplement: Supplementary file 1 — Supplementary [file 41598_2017_5240_MOESM1_ESM.pdf]

Integrated regulatory network reveals the early salt tolerance mechanism of *Populus euphratica*

Jiafei Chen[1,†], Jin Zhang[1,†], Jianjun Hu[1], Wenwei Xiong[2], Chunguang Du[2,*], Mengzhu Lu[1,*]

1. State Key Laboratory of Tree Genetics and Breeding, Key Laboratory of Tree Breeding and Cultivation of the State Forestry Administration, Research Institute of Forestry, Chinese Academy of Forestry, Beijing 100091, China

2. Department of Biology, Montclair State University, Montclair, NJ 07043, USA

† These authors contributed to this work equally.

* To whom correspondence should be addressed: email lumz@caf.ac.cn (M.L.) and duc@mail.montclair.edu (C.D.); phone +86 10 6288 9606; fax +86 10 6287 2015.

Table S1. Differentially expressed genes (DEGs) between the treated samples and control samples.

Table S2. DEGs among the treated samples during the time-course salt stress.

Table S3. Enriched GO-terms of DEGs in 20 clusters identified by K-means method (Fig. 4).

Table S4. Annotation of DEGs in the regulatory network underlying the co-regulated genes in *P. euphratica* (Fig. 6).

Table S5. Annotation of nodes in the integrated salt response pathway network (Fig. 7).

Table S6 Expression of DEGs putatively regulated by microRNAs.

Briefly 366 non-redundant transcripts were identified as putative targets of 331 miRNAs with a variety of correspondence, via the online tool psRNATarget using the default parameters. n.s. means no significant change was observed.

Table S7. Expression of target genes that have been proved to be regulated by miRNAs based on previous studies.

Table S8. The overlapped DEGs between our results and previous salt stress results.

The expression patterns of overlapped DEGs between our results and the results from previous studies. The genes were matched with the best homologous *P. trichocarpa*

gene ID based on BLASTn. The overlapped DEGs were marked as “common (Y)” in each comparison. “n.s.” means no significant changes were detected.

Table S9. The selected 38 genes and their primers used for qRT-PCR validation.
